# Nursing Training for Early Clinical Deterioration Risk Assessment: Protocol for an Implementation Study

**DOI:** 10.2196/47293

**Published:** 2023-10-17

**Authors:** Laura Bacelar de Araujo Lourenço, Mariana de Jesus Meszaros, Michele de Freitas Neves Silva, Thaís Moreira São-João

**Affiliations:** 1 School of Nursing University of Campinas Campinas Brazil; 2 College of Nursing University of Rhode Island Kingston, RI United States

**Keywords:** nursing, hospitalization, clinical deterioration, patient safety, early warning score, education

## Abstract

**Background:**

During the hospitalization period, it is possible to observe considerable changes in the vital parameters of patients, which may require emergency interventions or intensive treatment. The alteration of signs and symptoms that lead to physiological instability that can worsen the clinical picture with progression to shock, respiratory failure, or cardiorespiratory arrest is currently defined as clinical deterioration. Identifying signs of clinical deterioration at an early stage can lead to substantial decreases in mortality rates, the need for emergency interventions, and unscheduled treatments in intensive care units. Identifying and appropriately referring patients who show signs of clinical deterioration can be facilitated by applying early warning systems that provide rapid responses. The nursing team is usually the first to identify clinical changes in patients. Although the literature demonstrates that early recognition of clinical deterioration is the key to early intervention and leads to better outcomes, we only sometimes pursue the most appropriate intervention.

**Objective:**

This study aims to implement and evaluate an evidence-based professional training program designed for nurses and coordinated by a nurse using the “just-in-time” methodology and the National Early Warning Score 2 (NEWS2) to assess the risk of early clinical deterioration and appropriate referral in inpatient units of a public university hospital in southeastern Brazil.

**Methods:**

This intervention protocol is structured according to the recommendations of the SPIRIT (Standard Protocol Items: Recommendations for Interventional Trials) Declaration 2013. The type of training to be offered, “Just-in-Time Training,” consists of a teaching modality that facilitates the delivery of a time-based and work-based education, with greater emphasis on providing on-the-job learning as needed. A qualitative stage will also be conducted through focus groups and interviews with nurses to verify the factors that influence the professional practice related to the early evaluation of the clinic. A script of previously tested questions will guide and standardize the different groups. The data will define the intervention’s elements: the strategy, the type of training, the location, the teaching methodology, and the teaching material.

**Results:**

The study has received authorization from the ethics committee, and participants will be recruited in July 2023. Data collection should be completed in October of the same year. The results obtained at the end of this research will be shared with the participating nursing team through the presentation of reports. In addition, the research results will be submitted to scientific journals and presented at international scientific conferences.

**Conclusions:**

This study will support nurses and possibly other clinicians to improve their approach to early recognition of clinical deterioration in patients.

**Trial Registration:**

Brazilian Registry of Clinical Trials RBR-5hq9y3k; https://ensaiosclinicos.gov.br/rg/RBR-5hq9y3k

**International Registered Report Identifier (IRRID):**

PRR1-10.2196/47293

## Introduction

A patient’s clinical deterioration originates from physiological changes detected by monitoring vital signs, which can indicate early or potentially critical patients who need special monitoring in the wards [1,2]. Such changes can indicate a greater probability of cardiorespiratory arrest and lead to unplanned hospitalizations in intensive care units and death [3]. If there is a delay in the identification of these patients, it will result in delayed intervention and, therefore, in the increase of hospital mortality. The early identification of alterations in vital signs can facilitate greater efficiency in the care provided to the patient because the reduction in morbidity leads to a reduction in the length of hospital stays and lower expenditure on health, which can guarantee a better quality of care [1,4].

The nursing team is usually the first to identify clinical changes in patients. The nursing team comprises a diverse group of health care professionals collaborating to deliver patient care. This team typically includes registered nurses, licensed practical nurses or licensed vocational nurses, certified nursing assistants, nurse practitioners, nurse managers or supervisors, clinical nurse specialists, and additional support staff. These professionals possess specialized skills and training, allowing them to provide comprehensive care, administer treatments, monitor patient conditions, and fulfill administrative responsibilities. The composition of the nursing team may vary based on health care settings and specific patient needs.

The evaluation of vital signs is a routine activity of the nursing team in the hospital and is of extreme importance as it tracks the evolution of the clinical picture and the individual’s health and can predict clinical deterioration. Intrinsically, nursing is linked to patient surveillance and monitoring changes that may indicate warning signs, and these factors are interlinked with survival outcomes in cases of in-hospital cardiorespiratory arrest. Therefore, the nursing team can predict these events early to increase patient safety and prevent this clinical deterioration [5].

Early recognition of clinical deterioration is the key to early intervention and can lead to better patient outcomes [5]. Early warning systems are designed to detect changes and activate rapid response teams to minimize complications resulting from the degradation of the patient’s clinical condition before reaching a critical state of complex regression, which can be fatal [6]. Various systems have been described in the literature that use simple physiological parameters and routine assessments performed daily by nurses to manage the risk of degradation of the patient’s clinical status by adapting the level of care they need [7]. These systems are used to identify and respond efficiently to the needs of patients in an acute situation or phase [8].

Considering the PICOT (Population, Intervention, Comparison, Outcome, and Time) approach [9], this training was designed with nurses working in a hospital setting as the population of interest, the implementation of a professional training program for early clinical deterioration risk assessment as the intervention, the standard practice without the professional training program (ie, pretraining and posttraining) as the comparison, the improved identification and appropriate referral of patients showing signs of clinical deterioration as the outcome, and before and after the implementation of the professional training program as the timeframe. Therefore, the following research question guided the development of this training: “In nurses working in a hospital setting, does the implementation of a professional training program for early clinical deterioration risk assessment (compared to standard practice without the program) improve the identification and appropriate referral of patients showing signs of clinical deterioration?”

Considering this research question and given the options available in the literature of instruments that aim to facilitate the assessment of clinical deterioration, we decided to develop a training project for nurses to assess early clinical deterioration in a university hospital through the use of an alert scale designed for early identification of signs of clinical deterioration in patients. This protocol aims to implement and evaluate an evidence-based professional training program designed for nurses and coordinated by a nurse using the “just-in-time” methodology and the National Early Warning Score 2 (NEWS2) to assess the risk of early clinical deterioration and appropriate referral in inpatient units of a public university hospital in southeastern Brazil.

## Methods

This study was structured according to the recommendations of the SPIRIT (Standard Protocol Items: Recommendations for Interventional Trials) Declaration 2013 (Multimedia Appendix 1) [10].

### Trial Design and Settings

A quasi-experimental study of the type before and after, with a single arm, will be conducted with all nurses from inpatient units of a tertiary-level university hospital in the interior of the southeast of São Paulo to implement a training program to assess the risk of early clinical deterioration. This study aims to demonstrate causality between the proposed intervention and the nurses’ ability to use the NEWS2 tool to assess the patient’s clinical deterioration and provide appropriate referrals. Quasi-experimental designs are often used when assigning participants randomly to different groups is not feasible or ethical. This allows for intragroup comparison of outcomes (ie, comparing the pretraining and posttraining results in the same group). In this study, having only a subset of the staff receiving the training would not be feasible. Although a quasi-experimental design does not offer the same level of control as a randomized controlled trial, it can provide valuable insights into the effects of the training program because it offers a real-world evaluation of the intervention’s impact within the specific setting and considers the practical constraints of implementation.

The trial was registered in the Brazilian Registry of Clinical Trials (ReBEC) with the number RBR-5hq9y3k. For the design of the method, the limits of the Guideline for Reporting Evidence-Based Practice Educational Interventions and Teaching (GREET) [11] were respected.

### Eligibility Criteria

All assisting nurses allocated to the university hospital’s clinical and surgical hospitalization units in the interior of São Paulo will be included. During the data collection period, nurses who are inactive or on health or maternity leave or vacation will be excluded because they cannot be invited to participate in the training due to labor legislation issues.

### Intervention

The type of training to be offered, known as “Just-in-Time Training” (JITT), consists of an education modality that facilitates the delivery of time-relevant and work-based education, with a greater emphasis on teaching provision at work as needed [12]. JITT is a training approach that focuses on providing relevant and specific information to individuals when they need it to perform a task or solve a problem and is designed to provide immediate and targeted support, allowing nurses to acquire knowledge or skills quickly and efficiently. JITT is often used in contexts where time is limited and there is a need for spontaneous and on-demand learning. The effectiveness of the JITT method stems from several factors, such as punctuality, contextualization, and flexibility. By providing nurses with the correct information at a convenient time and place, JITT increases learning efficiency, minimizes continuums, and empowers individuals to quickly acquire the knowledge and skills they need to perform effectively in their specific tasks or problem-solving scenarios [12].

The JITT approach to recognizing and responding to patient deterioration has theoretical and educational relevance in its design. Education takes place in the clinical setting using real meetings with patients to guide the content. This approach is closely aligned with learning through a theory of practice, as the literature recommends [13]. In this perspective, workplaces are viewed as learning environments; optimizing the experiences provided by health workplaces, increasing learning potential, and promoting the involvement of workers can improve workers’ ability to respond to future occupational challenges.

The training program developed for nurses was based on the intervention mapping [14] protocol that describes the path from problem identification to problem solution or mitigation. Each of the six stages of intervention mapping comprises several tasks, integrating theory and evidence. Completing tasks in one step creates a product that drives the subsequent step. Completing all steps serves as a model for designing, implementing, and evaluating an intervention based on theoretical, empirical, and practical information.

#### Needs Assessment: A Theoretical and Experiential Approach

The needs assessment will be gathered by reviewing the literature on nurses’ knowledge and use of early deterioration tools. To use the experiential approach, a qualitative step will be conducted through the realization of focus groups and interviews with nurses from inpatient units to verify the factors that influence professional practice related to the early assessment of clinical deterioration. A script of previously established questions will guide and standardize the different groups (Textbox 1). The invitation to nurses will be made through a direct approach by verbal invitation from the researcher herself.

The interviews and focus groups will be based on a pre-established script (Textbox 2). All groups will have their audio recorded for future analysis, with the saturation criterion as a parameter for the decision to stop collection. The data collected in this phase will be evaluated through content analysis.

**Presentation of the project in the focus groups.**

- Team presentation: 3 nurses.
- Aims: Invite nurses to participate in a project offering training on early assessment of clinical deterioration.
- Planning: After agreeing and signing the consent form, the nurses will participate in a 30-minute focus group meeting. Six months after the meeting, training will be offered. Those who participate will receive feedback and a certificate.
- Setting: Nursing Department, University Hospital.

**Script to guide focus groups.**

- Do you know what early assessment of clinical deterioration in patients is?
- Do you usually perform an early assessment of the clinical deterioration of patients admitted to the wards? If so, how?
- For you, what is the importance of this assessment in your daily work in the ward?
- Do you use data from early clinical deterioration assessment to guide the care plan of patients under your supervision? If so, what data? How?
- Do you think it is feasible to carry out an early assessment of the clinical deterioration of patients admitted to the wards?
- In your opinion, what factors can favor the early assessment of clinical deterioration of patients admitted to the wards?
- In your opinion, what factors can hinder the early assessment of clinical deterioration of patients admitted to the wards?
- How would you like to receive this training? Reflect and give your opinion on the best strategy, the type of training, location, teaching methodology to be used and teaching material.

#### Establishment of Training Objectives

Following the needs assessment stage, we will establish the training objective. The most appropriate content and training strategies for the target population and context should be identified at this stage. The data obtained will allow us to define the elements of the intervention, including the strategy, type of training, location, teaching methodology, and teaching material.

#### Intervention Strategy Proposal

In this stage, the content to be addressed, the methods design, and the strategies and means of communication will be defined.

#### Pretest

This phase will consist of a pretest of the intervention applied to a population subsample to verify operational aspects of the chosen content, strategy, and means of communication. This step aims to assess the adequacy and feasibility of the proposed intervention.

#### Preintervention Assessment

In this step, a questionnaire [15] will be used to assess the theoretical content related to each participating nurse’s early assessment of clinical deterioration to compare the pretraining and posttraining results. The Brazilian version of the rapid response team (RRT) survey, which aims to explore nurses’ knowledge and perceptions of RRTs, was translated and adapted into Brazilian Portuguese and validated (LBdA Lourenço and TM São-João, unpublished data). This instrument has 3 parts: part 1 contains 4 hypothetical case studies; in part 2, respondents must evaluate each factor according to its degree of importance; and in part 3, participants must include their basic information regarding characteristics and work history.

Although this tool has been widely used globally, no studies have been published on its psychometric properties. Assessing the psychometric properties of a measurement tool is relevant for ensuring its validity, reliability, and suitability for data collection in most research contexts. However, there are situations where a psychometric evaluation may be unnecessary, such as for well-established and widely used tools, especially those that do not measure psychosocial constructs (eg, the RRT survey). Once the tool has been extensively used, widely accepted, and has a solid empirical foundation in the literature, conducting a full psychometric assessment may not be necessary [16].

#### Implementation of the Intervention

The planned intervention will be implemented via the chosen method, and the participants will be informed in advance to allow for planning and effective participation.

#### Postintervention Assessment

In this step, the Brazilian version of the RRT survey will be reapplied to compare the pretraining and posttraining results and evaluate the effectiveness of the intervention.

#### Assessment of Suitability, Acceptability, and Quality of the Intervention

The nurses’ assessment regarding the suitability, acceptability, and quality of the proposed intervention will be assessed through a self-answered, open-questions questionnaire. This questionnaire will have its content submitted for validation by specialists.

#### Criteria for Discontinuing or Modifying Allocated Interventions for a Given Trial Participant

Participants will be discontinued for not attending scheduled meetings or ending participation in the study during the intervention or data collection phase.

#### Strategies to Improve Adherence to Intervention Protocols and Procedures for Monitoring Adherence

An SMS text message will be sent to participants’ telephones a day before the scheduled appointment, reminding them to attend the training.

#### Relevant Interventions That Are Permitted or Prohibited During the Trial

During the intervention, participants must not engage in any other training regarding clinical deterioration.

### Outcomes

A form for analyzing the sociodemographic profile will be applied to nurses with the following variables: age, sex, education level, time of training, place, and time of work in the unit.

The effect of evaluating the theoretical content related to the early assessment of clinical deterioration by each participating nurse will be considered to compare the pretraining and posttraining results.

### Sample Size and Sampling

The minimum sample size was calculated according to the stages of data collection in the pretest and posttest. To evaluate the responsiveness of part 1 of the RRT survey comparing the scores from the two evaluation periods, a paired student *t* test was used. In this calculation, a significance level of 5% was assumed with a test power of 80% and an effect size of 0.50, which, according to Cohen [17], can be considered an effect size of medium degree. The calculation resulted in a minimum sample of 34 participants. To carry out this sample calculation, G*Power 3.1.9.2 software was used [18,19].

To assess the internal consistency of part 2 of the RRT survey, we referred to the recommendation proposed by the COSMIN (Consensus-Based Standards for the Selection of Health Measurement Instruments) initiative that at least 50 participants are necessary for a sample to be considered adequate when this measurement property is evaluated [20].

To evaluate assertiveness in filling out the NEWS2 before and after the intervention, the same methodology was proposed by the COSMIN initiative that at least 50 participants are necessary [20]. Thus, 50 participants will be included in the study to meet all the proposed objectives.

### Recruitment

The human resources records of each eligible participant will be accessed through a list of registered nurses in floor units. Participants who engage in the study will be scheduled for a baseline appointment. On the appointed day, the interviewer will personally invite the participant and both will sign two copies of the research consent form.

### Randomization and Allocation Concealment

Considering the nature of a JITT, no randomization will be conceived. All nurses allocated in floor units will be sequentially included according to their availability, schedule, and willingness to participate.

### Implementation

The lead investigator will implement intervention strategies.

### Blinding

This is not a blind study as both the lead investigator and participants will know they are receiving an intervention.

### Data Collection

The experiment will be offered from July to December 2023 and include nurses who work in the clinical and surgical floor units.

Initially, a meeting will be held with the managers of the inpatient units to present the training program. Then, nurses will be recruited through an individual invitation to be offered by the researcher in the work units. After acceptance by the participants, a roster will be set up, together with the managers of the units, to assist in the organization of work rosters so that every position is covered when the nurses leave to participate in the training. Several training sessions will be made available in the morning, afternoon, and evening to carry out the training within the working hours of each nurse and according to what was described in the JITT method. The training sessions are structured to be 1 hour long and include the following steps: training presentation, signing of the informed consent form, application of the pretest, training, application of the posttest, and final evaluation. All nurses will participate in a single session and receive a participation certificate after completing all the steps described. The training will be carried out in a training room within the hospital.

Content analysis will be carried out during the qualitative phase (focal group). The most appropriate contents and strategies for nurses, as defined by them, will be combined to determine the elements of the intervention. Regarding the web-based questionnaire and the RRT survey, both of which are quantitative instruments, any increase in the score from the pretest to posttest will represent an improvement and, consequently, indicate the effectiveness of the intervention.

### Data Management

The gathered data will be compiled in a spreadsheet using Excel 2010 (Microsoft Corporation) using double entry. In this spreadsheet, numerical encodings will be created for categorical variables. Quantitative variables will be inserted as collected. To avoid erroneous double entries or differences in the created codes, preset values for categorical variables will be used. The spreadsheet containing the data will be password-protected and solely accessible by the lead investigator.

### Data Analysis

The data spreadsheet will be imported into SAS 9.4 software (SAS Institute). The results of the focus groups, as well as the preintervention and postintervention assessment questionnaires, will be submitted for content analysis.

### Statistical Analysis

Qualitative variables will be described by calculating frequencies and percentages, and quantitative variables through position measurements and dispersion.

Comparisons between the two time periods concerning the score of the NEWS2 instrument and part 1 of the RRT survey instrument will be carried out using paired Student *t* tests or paired Wilcoxon tests [21], according to the data distribution. The distribution of data will be evaluated using the Shapiro-Wilk test.

The internal consistency of part 2 of the RRT survey instrument will be assessed using Cronbach α coefficient [22]. This coefficient ranges from 0 to 1, where values >.7 indicate that the measurements are reliable [23].

The statistical software SAS and SPSS (version 28; IBM Corp) will be used to carry out the analysis with a significance level of 5%.

### Data Monitoring

No monitoring committee was established for this study because we understand it will bring minimal risk to participants, as described in the section Harms below. In addition, the protocol may be terminated early due to these minimal predicted risks.

### Harms

For this study, small risks and discomforts to participants are foreseen. Participants may feel uncomfortable during the encounters or while answering any questions about clinical deterioration, and they may need to know the answer.

### Auditing

The following audit procedures will be considered: (1) compliance audits, by assessing the extent to which nurses are complying with the prescribed training protocol (ie, reviewing records, documentation, and training logs to verify if nurses have completed the required modules, attended relevant sessions, or fulfilled certification requirements); (2) knowledge assessments, by assessing nurses’ understanding of the content; (3) skills evaluations, by observing and assessing nurses’ performance of the specific skills or procedures required through direct observation, simulated scenarios, and practical assessments; (4) feedback surveys, to gather feedback from nurses about their training experience; and (5) quality assurance reviews of training materials, resources, and procedures, to ensure they align with evidence-based practices.

### Ethical Considerations

The local Research Ethics Committee approved the study (Certificate of Submission for Ethical Review n. 37499220.9.0000.5404). All enrolled participants will sign the informed consent form, according to Resolution 196/96 of the National Health Council, when instructed about anonymity and the freedom to withdraw their consent at any time during the research. It should be noted that the database will not be sent abroad for analysis. The study was also registered in ReBEC (RBR-5hq9y3k).

The lead investigator shall keep research forms for 5 years, at which time they shall be incinerated. A multimodal dissemination strategy will be used to share findings with multiple stakeholders. Strategies will include scientific publications and presentations in the form of an expository and oral class in previously scheduled meetings with hospital managers and the participating nursing team, with the results obtained at the end of the training program implementation.

## Results

Data collection will start in July 2023 with results expected in December of the same year. This study aims to describe a protocol for a professional training program developed for nurses so that they can implement early assessment of the risk of clinical deterioration and offer appropriate referrals. The protocol will also influence nursing managers and nurses in elaborating protocols and clinical practice because, as the first team to identify changes in the status of patients, the importance of nursing in detecting clinical deterioration is recognized.

The results obtained at the end of this research will be shared with the participating nursing team through the presentation of reports. In addition, we will submit the research results to scientific journals and present them at international scientific conferences.

## Discussion

### Innovative Protocol for Nurse Training in Early Detection of Clinical Deterioration

This study will describe a protocol for a professional training program designed for nurses to implement early risk assessment of clinical deterioration. This protocol is unprecedented and of great relevance, as some studies [24,25] have already demonstrated that the implementation of clinical deterioration evaluation directly impacts nurses in three areas of competence: (1) evaluation and care of patients (through the detection of clinical deterioration and development of skills and knowledge); (2) referring patients (through knowledge of the language and lines of communication in the referral process and deciding whether to seek help); and (3) coping experiences. Studies have also demonstrated that most patients who required hospitalization in intensive care units or who experienced cardiopulmonary complications exhibited signs of clinical deterioration hours before the event; more than 80% of these patients could be identified approximately 24 hours before these serious adverse events [26]. In this way, identifying patients who can benefit from intensive care units becomes crucial [26].

The development of this proposal is expected to implement a pioneering early warning system with Unicamp Clinical Hospital to increase the quality of care, improve indicators related to patient safety, and decrease the preventable hospital mortality rate.

### Limitations

Some limitations pertaining to the study’s design and methodology exist. The limited generalizability is a concern, as the study will be conducted in a specific public university hospital in southeastern Brazil, potentially limiting the applicability of the findings to other health care settings or regions. Potential selection bias is another limitation, as the recruitment process will not be randomized. The data collection period may restrict the gathering of a comprehensive and long-term data set.

The second set of limitations revolves around data collection and analysis. The qualitative stage involving focus groups and interviews with nurses introduces the possibility of self-reporting bias, where participants may provide socially desirable responses, or their recollections may not accurately reflect their actual practices. Finally, it is not possible to control or account for potential confounding factors, such as patient demographics or other interventions that may influence the outcomes.

Despite the acknowledged limitations, this paper provides a valuable contribution by outlining the design of a nurse-led intervention program that other health care professionals can replicate. The structured and systematically mapped intervention presented in this study offers the potential for improved resource allocation and results optimization. By enhancing nurses’ ability to detect early signs of clinical deterioration and facilitating prompt and accurate interventions, this program holds promise for improving patient outcomes and increasing the likelihood of positive treatment outcomes.

Some studies have demonstrated the effectiveness of using intervention mapping in nursing in planning and implementing interventions and evaluating results. The research subjects reported that the interventions allowed a better understanding of the studied models [27,28].

### Conclusions

This study aims to contribute to advancing nursing practices in the context of clinical deterioration among inpatients. The intervention proposed in this research is grounded in established theoretical principles and is characterized by its cost-effectiveness and ease of implementation. Specifically tailored for adult patients in inpatient units, this program holds the potential to substantially enhance nurses’ ability to accurately diagnose and promptly intervene in cases of early clinical deterioration. The paper introduces an evidence-based professional training program designed for nurses and led by a nurse, implementing a risk assessment protocol for early clinical deterioration within the inpatient units.

## Appendix

Multimedia Appendix 1 SPIRIT 2013 checklist.

## Abbreviations

COSMIN: Consensus-Based Standards for the Selection of Health Measurement Instruments
GREET: Guideline for Reporting Evidence-Based Practice Educational Interventions and Teaching
JITT: Just-in-Time Training
NEWS2: National Early Warning Score 2
PICOT: Population, Intervention, Comparison, Outcome, and Time
ReBEC: Brazilian Registry of Clinical Trials
RTT: rapid response team
SPIRIT: Standard Protocol Items: Recommendations for Interventional Trials

## Data Availability

The data sets generated and analyzed during this study are available from the corresponding author upon reasonable request. Data and metadata will be made available in the University of Rhode Island Research Data Repository (DigitalCommons@URI) after the completion of the project and for 10 years.
