# Supplementary material for: Nursing Training for Early Clinical Deterioration Risk Assessment: Protocol for an Implementation Study
Source: JMIR Res Protoc. 2023 Oct 17;12:e47293. doi: 10.2196/47293 (PMC10618875; doi:10.2196/47293)
Supplement: Multimedia Appendix 1 [file resprot_v12i1e47293_app1.docx]

**SUPPLEMENTARY FILE 1: SPIRIT 2013 CHECKLIST**

Supplementary File 1 shows recommended items to address in a clinical trial protocol and related documents, based on the SPIRIT (Standard Protocol Items: Recommendations for Interventional Trials) checklist.

**Supplementary File 1**: SPIRIT 2013 Checklist

| **Section/Item** | **Item No.** | **Description** |
| --- | --- | --- |
| **Title** | 1 | Nursing Training for Early Clinical Deterioration Risk Assessment: Protocol for an Implementation Study. |
| **Trial registration: ‑‑Registry identifier** | 2a | It is registered in the Brazilian Clinical Trials Registry and the unique identifier is RBR-5hq9y3k. |
| **‑‑WHO registration** | 2b | N/A |
| **Protocol version** | 3 | 7/20/2022, Version 1 |
| **Funding** | 4 | This research is funded by:  1 - Coordination for the Improvement of Higher Education Personnel (CAPES) – PhD Scholarship for LBAL;  2 – University of Rhode Island – Internal Funds for TMSJ.  3 - This is a project previously included in the project entitled “Health promotion and rehabilitation in chronic diseases: from basic research to the translation of knowledge”, of the School of Nusing/Unicamp, which receives support from the Institutionalization Program of Internationalization - CAPES - PrInt-Unicamp; |
| **Roles and responsibilities:  ‑‑Authors** | 5a | All authors have the same responsibility for the manuscript and declare that they have no conflicts of interest. |
| **‑‑Trial sponsor** | 5b | N/A |
| **‑‑Funders** | 5c | The funders are not involved in the collection, analysis and interpretation of data; in the writing of the report; or in the decision to submit the paper for publication. |
